# Validation of a questionnaire on the use of Interactive Response System in Higher Education

**DOI:** 10.1590/1518-8345.3374.3418

**Published:** 2021-06-28

**Authors:** Ángel Custodio Mingorance-Estrada, Juan Granda-Vera, Gloria Rojas-Ruiz, Inmaculada Alemany-Arrebola

**Affiliations:** 1 University of Granada, Department of Didactics and School Organization, Melilla, ES, Spain.; 2 University of Granada, Department of Didactics of Corporal Expression, Melilla, ES, Spain.; 3 University of Granada, Department of Developmental and Educational Psychology, Melilla, ES, Spain.

**Keywords:** Education, Higher Education, Learning, Validation Study, Surveys and Questionnaires, Technology Education, Educação, Ensino Superior, Aprendizagem, Estudos de Validação, Pesquisas e Questionários, Tecnologia Educacional, Educación, Educación Superior, Aprendizaje, Estudios de Validación, Encuestas y Cuestionarios, Tecnología Educacional

## Abstract

**Objetivo:**

this study aims to design and validate a questionnaire to measure the students’ perception of the use of IRS as a technopedagogical resource in the classroom.

**Method:**

a 24 items questionnaire (Interactive Response System for the Improvement of the Teaching-Learning Process) was designed *ad hoc* for this research and applied to 142 university students.

**Results:**

both the exploratory and confirmatory factorial analysis yielded 3 dimensions: classroom environment, teaching-learning processes and learning assessment. The results obtained both in reliability (Cronbach’s alpha= 0.955) and in the Confirmatory Factor Analysis (χ2/df=1.944, CFI=0.97; GFI=0.78; RMR=0.077; RMSEA=0.08) reveal highly satisfactory indices.

**Conclusion:**

statistical analyses confirm that this instrument is a valid, reliable, and easy-to-apply tool for professors to evaluate the student perception of student-centred learning.

## Abstract (pt)

**Objetivo:**

o presente estudo visa conceber e validar um instrumento para medir a percepção dos estudantes universitários sobre a utilização dos Dispositivos Móveis Interativos (MIR) como recurso tecnológico-pedagógico na sala de aula.

**Método:**

foi aplicado o questionário “*Mandos Interactivos de Respuesta para la Mejora del Proceso de Enseñanza-Aprendizaje*”, criado ad hoc para esta investigação, composto por 24 itens e aplicado a 142 estudantes universitários.

**Resultados:**

tanto a análise fatorial exploratória quanto a confirmatória desembocam em 3 dimensões: ambiente de sala de aula, processos de ensino-aprendizagem e avaliação. Os resultados obtidos tanto na confiabilidade (alfa de Cronbach 0,955) como na análise fatorial confirmatória (χ^2^/gl=1,944, CFI=0,97; GFI=0,78; RMR=0,077; RMSEA=0,08) produzem índices altamente satisfatórios.

**Conclusão:**

análises estatísticas mostram que este instrumento é válido, confiável e fácil de aplicar para os professores avaliarem a experiência estudantil de aprendizagem centrada no aluno.

## Abstract (es)

**Objetivo:**

el presente estudio tiene como objetivo diseñar y validar un cuestionario que permita medir la percepción de los estudiantes universitarios del uso de los MIR como recurso tecnopedagógico en el aula.

**Método:**

se aplicó el cuestionario Mandos Interactivos de Respuesta para la Mejora del Proceso de Enseñanza-Aprendizaje, creado *ad hoc* para esta investigación, que está formado por 24 ítems, aplicado a 142 estudiantes universitarios.

**Resultados:**

tanto el análisis factorial exploratorio como el confirmatorio arrojan tres dimensiones: ambiente de clase, los procesos de enseñanza-aprendizaje y la evaluación. Los resultados obtenidos tanto en la fiabilidad (alfa de Cronbach 0.955) como en el análisis factorial confirmatorio (χ^2^/gl=1.944, CFI=0.97; GFI=0.78; RMR=0.077; RMSEA=0.08) arrojan índices altamente satisfactorios.

**Conclusión:**

los análisis estadísticos muestran que este instrumento es una herramienta válida, fiable y de fácil aplicación para el docente que le permitirá evaluar la experiencia del alumnado sobre el aprendizaje centrado en el estudiante.

## Introduction

Higher Education must establish a space for the exchange of practical experiences that promotes knowledge and research within the common framework of the European and Ibero-American Higher Education Area^(^ 1 ^-^ 2 ^)^.

The implementation of student-centered learning is oriented to establish a model that effectively integrates technology with knowledge of didactic mediation, evolving towards the Technological Pedagogical and Content Knowledge Model (TPACK). This system combines disciplinary, pedagogical and technological knowledge, but always considering the context in which it intervenes^(^ 3 ^-^ 4 ^)^, and increasing the interaction between professors and students within a critical dialogical approach^(^ 2 ^)^.

Some authors consider necessary to establish didactic knowledge through the relationship between different types of knowledge (coming from the own discipline, general pedagogy and students) and the professor’s biography^(^ 5 ^)^. Thus, during the nursing initial training, both the pedagogical aspects, including the implementation of Interactive Response System (hereinafter IRS) with remote answering devices to monitor students’ progress, and the involvement of expert professors lead to high-quality teaching, among other issues^(^ 6 ^)^.

In this regard, encouraging professors to integrate technology into the classroom is crucial, as highlighted in the Horizon Report^(^ 7 ^)^, since it will significantly impact on education in the coming years. To do this, university professors must use the technological tools they are familiar with, as well as access new technological resources to improve teaching processes^(^ 8 ^)^.

Similarly, technological changes in university professors follow a tendency and are not radical; they introduce those that are consistent with their teaching practices into the learning activities they normally carry out^(^ 9 ^)^.

In order to face these technological challenges, an inversion of learning process is required; students should be provided with materials in various formats, so that they can carry out preliminary work before arriving at the classroom, incorporating IRS to verify the improvements in the student centered-learning process^(^ 10 ^-^ 12 ^)^. IRS has been already integrates in some university classes ^(^ 13 ^)^. Thus, the available literature about Higher Education on the use of this technology in recent years focuses on the fields of Science, Technology, Engineering and Mathematics, Sociology, Humanities, Health (Medicine and Nursing), Business Administration and English language^(^ 13 ^-^ 14 ^)^.

The reviewed studies indicate that integrating IRS in university classrooms improves three main areas: the classroom environment, the teaching-learning processes and learning assessment^(^ 15 ^-^ 20 ^)^. Thus, defining the possibilities and limitations of this tool is increasingly important for the improvement of the quality of Higher Education^(^ 6 ^)^.

Concerning the *classroom environment* factor, IRS increases attendance^(^ 21 ^)^ and student participation^(^ 22 ^-^ 25 ^)^, resulting in a higher level of involvement during classes in comparison to the traditional methodology^(^ 23 ^,^ 26 ^-^ 27 ^)^. Within the learning factor, some studies establish that frequent and positive interaction makes classes more dynamic when using IRS^(^ 13 ^,^ 28 ^-^ 29 ^)^, promoting active learning^(^ 30 ^-^ 32 ^)^. In addition, attention^(^ 33 ^-^ 34 ^)^, concentration^(^ 35 ^)^ and memory^(^ 36 ^-^ 37 ^)^ are encouraged during the learning process. Extensive research suggests that a better performance is the result of the use of IRS, as some studies indicate^(^ 31 ^-^ 32 ^,^ 38 ^-^ 41 ^)^, although other studies do not find such an effect^(^ 42 ^)^. In relation to the assessment factor, the findings of the literature support the capacity of IRS as a tool for assessment and feedback^(^ 43 ^-^ 44 ^)^. It is considered that both students and professors benefit from the feedback they receive with the use of this educational technology^(^ 20 ^,^ 27 ^)^. All of this leads to a key learning process for the interaction of knowledge and know-how^(^ 45 ^)^.

The aim of this study is to design and validate this study aims to design and validate a questionnaire to measure the students’ perception of the use of IRS as a technopedagogical resource in the classroom.

## Method

The design was transectional and descriptive, as the data were collected in a single time in order to describe the phenomenon and analyze it at a certain time.

The research was carried out at Melilla Campus of the University of Granada (Spain), located in North Africa, whose students attend to the Health Sciences, Social and Legal Sciences, and Education and Sports Sciences schools. For this purpose, an intentional non-probabilistic sample was carried out. The selection criteria have been: firstly, professors who use technology in their classrooms, specifically interactive response devices. And secondly, the willingness of the students to participate in this study. Therefore, the sample comprises 142 students: 110 women (77.5%) and 32 men (22.5%). In relation to the academic year, 17 students are in first year (12%), 95 in second year (66.9%) and 30 in third year (21.1%).

To carry out this study, an *ad hoc* questionnaire was designed for this research, “Interactive Response System for the Improvement of the Teaching-Learning Process (IRS-ITLP)”.

In relation to the items of the IRS-ITLP, they were written after an extensive bibliographic review on the three factors highlighted above^(^ 21 ^,^ 46 ^-^ 47 ^)^. Despite of the absence of experts on this field, this process provides validity to the questionnaire items.

We started with 65 items grouped into categories^(^ 48 ^)^. After the analysis of the items, we selected 35 items that were grouped into the three dimensions: learning environment, process and assessment. Regarding the questionnaire response format, a Likert type scale was used, with 5 response alternatives, ranging from 1, totally disagree, to 5, totally agree.

The research was oriented to different degrees of the University of Granada (Spain), all of them being subjects concerning the basic formation of the students. For this purpose, the collaboration of the teaching staff was requested to participate as volunteer in this project and to integrate IRS in their classes.

The use of the IRS in these basic training subjects was carried out throughout the semester of the 2016-17 academic year, before, during and at the end of the classes. At the end of the semester, the IRS-ITLP questionnaire was applied the last week of the semester, with a duration of approximately 15 minutes, to find out the perception of the experience. Students were asked to agree to participate in this experience voluntarily and anonymously, following the rules of the Committee on Publication Ethics (COPE).

The statistical software SPSS version 20.0 has been used for the statistical processing of the data. To know the reliability of each group of items, Cronbach’s alpha was used, and for the validity of the questionnaire, an Exploratory Factor Analysis was carried. For the Confirmatory Factor Analysis, the program LISREL 8.8 was used.

## Results

Firstly, the reliability of the IRS-ITLP questionnaire consisting of 35 elements was analyzed using Cronbach’s alpha internal consistency coefficient, which was 0.965. Although this index was high, we proceeded to eliminate those items whose item-total correlation was inferior to 0.20. Finally, the questionnaire was made up of 24 items with a α=0.955, showing homogeneity indexes ranging from 0.42 to 0.85.

Subsequently, the means, standard deviations, asymmetry, and item-total correlations of each of the items were obtained. As can be seen in Table 1, the asymmetry is negative in all items, which shows a greater concentration of responses corresponding to the high scores in those items.

**Table 1:** Descriptive values of the items in the IRS-ITLP questionnaire. Granada, Spain, 2017

Nº	Items	M*	SD^†^	Asymmetry	Correl. item-total
1	I am more focused during the classes since the implementation of IRS	3.40	1.15	-0.477	0.689
2	Thanks to IRS, I measure if I am following correctly the contents of the subject during the classes	3.81	1.01	-0.658	0.619
5	During my experience with the IRS I have a good time learning	3.52	1.18	-0.559	0.566
9	IRS is used to find out the initial knowledge of the students	3.54	1.258	-0.595	0.488
10	The use of IRS is carried out by experienced professors to provide good feedback	3.97	0.891	-0.616	0.610
14	The use of IRS helps me to develop my comprehension on the contents I am working on	3.59	1.162	-0.666	0.639
15	The use of the IRSs makes the classes enjoyable and dynamic	3.82	1.119	-0.877	0.656
18	The use of IRS improves my learning performance	3.76	1.129	-0.653	0.762
19	The continuous use of the IRS increases my class attendance	3.70	1.266	-0.793	0.579
20	The use of IRS allows you to know and compare your colleagues' answers with your own answers	3.42	1.234	-0.646	0.441
21	The use of the IRS allows to correct mistakes or misunderstandings about the subject contents during the classes	3.71	1.121	-1.032	0.558
22	I am more interested in classes when using IRS	3.65	1.066	-0.698	0.768
24	I like the use of IRS as an attendance control	3.65	1.005	-0.605	0.699
29	The use of IRS improves motivation during classes	3.83	1.111	-0.825	0.753
30	The use of IRS allows active discussion of misconceptions to build knowledge	3.80	1.168	-0.771	0.792
35	The use of the IRS evaluates my comprehensive knowledge of the contents in each of the topics covered during the classes	3.99	0.971	-1.211	0.788
36	The use of IRS promotes regular study of the subject to be better prepared for classes	3.64	1.094	-0.762	0.715
42	The use of IRS allows you to be more confident when asking questions during classes	3.76	1.254	-0.739	0.787
46	The use of the IRS is done at the end of the classes to review the contents explained during the session	3.80	1.100	-0.736	0.772
47	The use of IRS makes the classes more pleasant and interactive compared to traditional classes	3.86	1.082	-0.804	0.769
48	The use of IRS improves your participation in classes behind anonymity	3.87	1.104	-0.773	0.712
53	The answers provided through the IRS increase my confidence in the classes after verifying that I answered correctly	3.44	1.164	-0.420	0.700
59	IRS provides valuable information to improve your learning process	4.09	1.017	-1.007	0.687
65	The use of IRS improves the understanding of the contents explained in class	3.87	1.160	-0.911	0.727

* M = Mean;

† SD = Standard deviation

Since any study about the factor analysis of the IRS-ITLP questionnaire had previously been published, before performing a Confirmatory Factor Analysis it was convenient to carry out an Exploratory Factor Analysis (EFA) to explore how the items are grouped into factors. To ensure that the data fit a factor analysis model, the data were subjected to the Kaiser-Meyer-Olkin test (KMO= 0.941) and to the Bartlett’s Test for Sphericity (c^2^= 2446.206; df= 300; *p*<0.001). The values indicate that a factor analysis is a suitable technique to structure the information contained in the matrix. The EFA reveals the existence of 3 factors that explain 61.61% of the total variance, being this proportion acceptable. In addition, the item communalities are above *h* ^2^=0.40, ranging from 0.421 “The continuous use of IRS increases my class attendance” to 0.791 “The use of IRS allows you to know and compare your colleagues’ answers with your own answers”.

Table 2 shows the factors, items, factor loadings and reliability of each dimension, as well as the interpretation of these factors. To determine the dimensions, the factorial loadings criterion has been followed, being the cutoff value 0.30^(^ 49 ^)^.

**Table 2:** Factors, items and loadings obtained in the Exploratory Factor Analysis of the IRS-ITLP. Granada, Spain, 2017

Alpha, Factor loadings	Items,factors and variance explained	1	2	3		h2*	α^†^
FACTOR 1: Learning environment							
**F1** 51.07%	15.The use of IRS makes the classes enjoyable and dynamic	0.759		0.625			0.926
	30. The use of IRS allows active discussion of misconceptions to build knowledge	0.748	0.303	0.736			
	48. The use of IRS improves your participation in classes behind anonymity	0.731		0.650			
	42. The use of IRS allows you to be more confident when asking questions during classes	0.685	0.403	0.673			
	53. The answers provided through IRS increase my confidence in the classes after verifying that I answered correctly	0.640		0.537			
	5.During my experience with IRS I have a good time learning	0.602	0.347	0.484			
	22. I am more interested in classes when using IRS	0.577	0.497	0.641			
	47. The use of IRS makes the classes more pleasant and interactive compared to traditional classes	0.562	0.491	0.644			
	24. I like the use of IRS as an attendance control	0.538	0.480	0.590			
	1. I am more focused during the classes since the implementation of IRS	0.483	0.560	0.564			
**FACTOR 2: Teaching-learning process**							
**F2** 5.47%	18. The use of IRS improves my learning performance	0.404	0.726	0.740			0.869
	9. The IRS is used to find out the initial knowledge of the students		0.725	0.550			
	2. Thanks to the IRS I measure if I am following correctly the contents of the subject during the classes		0.702	0.599			
	59. IRS provides valuable information to improve your learning process		0.628	0.512			
	10. The use of IRS is carried out by experienced professors to provide good feedback	0.324	0.576	0.483			
	46.The use of the IRS is done at the end of the classes to review the contents explained during the session		0.402	0.687			
	19. The continuous use of the IRS increases my class attendance		0.516	0.422			
**FACTOR 3: Learning Assessment**							
**F3** 5.07%	20. The use of IRS allows you to know and compare your colleagues' answers with your own answers			0.864		0.755	0.871
	36.The use of IRS promotes regular study of the subject to be better prepared for classes	0.403		0.678		0.686	
	21. The use of the IRS allows to correct mistakes or misunderstandings about the subject contents during the classes		0.361	0.641		0.576	
	35. The use of the IRS evaluates my comprehensive knowledge of the contents in each of the topics covered during the classes	0.604		0.523		0.715	
	29. The use of IRS improves motivation during classes	0.391		0.491		0.680	
	65. The use of IRS improves the understanding of the contents explained in class		0.332	0.486		0.602	

* h2 = Communality;

† α= Cronbach's alpha

Subsequently, the Confirmatory Factor Analysis was performed, and the 3-factors model was tested. The maximum likelihood estimation method was used to analyze the correlation matrix. The Goodness-of-Fit of the proposed model was evaluated using various indicators. The χ^2^/df (484.13/249) scores 1,944, a value that is within the acceptable standards. Moreover, the Root Mean Square Residual (RMR) is 0.077 and the Root Mean Square Error of Approximation (RMSEA) is 0.080, being both indexes considered acceptable since they are between 0.5 and .08^(^ 49 ^)^. The Goodness-of-Fit Index (GFI) and the Comparative Fit Index (CFI) (with values of 0.78 and 0.97 respectively) are within the tolerance limits. These results confirm that the 3-factor model fits the data, so the model can be maintained as a plausible explanation for the proposed dimensional structure.

**Figure 1:**
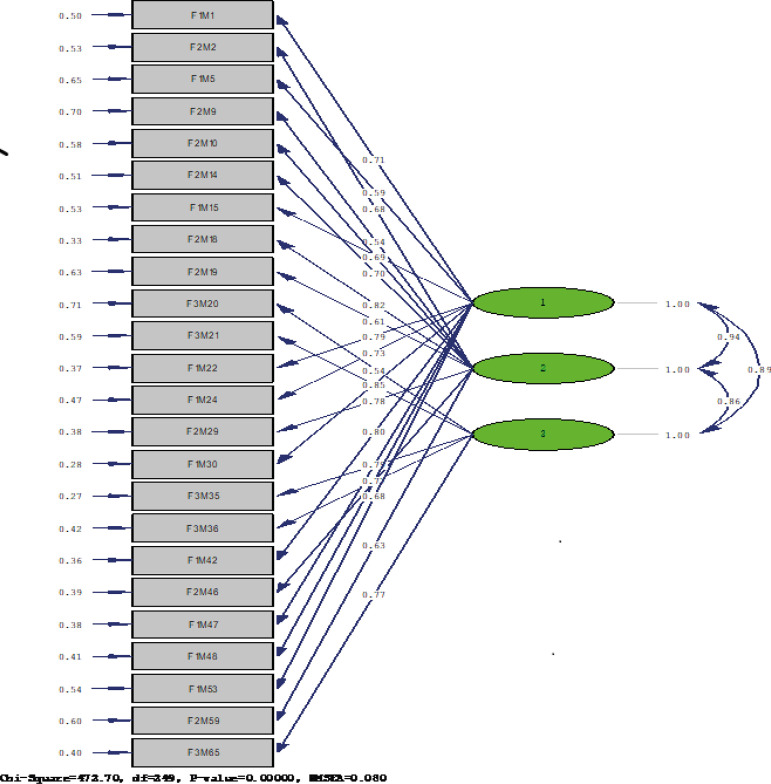
Confirmatory factor analysis of the questionnaire “Interactive Response System for the Improvement of the Teaching-Learning Process”

To test the reliability of the instrument, a Cronbach’s alpha test is carried out, obtaining a total value with a α= 0.955 and the dimensions that make it up, obtaining values that range from α=0.922 for factor 1, “Environment”, to α= 0.869 in factor 2 “Teaching-learning process”. These data show that the reliability of the questionnaire is good in all the factors, being lower in factor 3, “Assessment”. Although this statistic has been widely used in social research, it should be complemented with other analysis, such as the Composite Reliability Index (CF) and the Mean Extracted Variance (MEV). The results obtained are shown in Table 3, being in all cases acceptable.

**Table 3:** Composite Reliability and Mean Extracted Variance from the factors in the IRS-ITLP questionnaire. Granada, Spain, 2017

Factors	CF*	MEV^†^
F1: Learning Environment	0.955	0.544
F2: Teaching-learning process	0.930	0.508
F3: Learning assessment	0.863	0.540

* CF= Composite Reliability;

† MEV = Mean Extracted Variance

## Discussion

The aim of this study is the construction of a valid and reliable questionnaire to measure the use of IRS in university student-centred learning. The results provide empirical evidence of the validity of this intervention model using IRS, which allows university professors in general, and health sciences professors in particular, to transform the teaching-learning process. This model encourages and involves students in this process through a more active approach for 21^st^ century professors, who can measure students’ perception on the use of IRSs, an advance that really makes a difference.

Moreover, apart from a technopedagogical resource, it turns into a playful activity for students in a non-game context, being this model included in an emerging educational methodology called Gamification. In summary, this model provides the opportunity to drastically transform traditional classrooms so that it improves the classroom environment, the learning process and their academic performance in a playful and enjoyable way.

The IRS-ITLP questionnaire consists of 24 items that are grouped into 3 factors: Learning Environment, Teaching-Learning Process and Assessment. In relation to the quality of the items, which was measured through item-to-total correlation, the data indicates high rates ranging from 0.488 to 0.787. These values show a high internal consistency supporting the ideas that the items are correlated, and the scale is accurate. Furthermore, a descriptive analysis on the items show that there is negative asymmetry, which reveals that university students tend to agree with the questionnaire statements.

As for the reliability of the scale, the value for Cronbach ‘s alpha is 0.965, indicating a high reliability. These data are in line with the composite reliability indexes of the 3 factors comprising IRS-ITLP, which reach optimum levels: 0.955, 0.930 and 0.863 for Environment, Teaching-Learning Process and Assessment, respectively, being the minimum acceptable value 0.70.

Furthermore, the validity of the construct was tested through an Exploratory Factorial Analysis (EFA) and a Confirmatory Factorial Analysis (CFA). Firstly, the EFA was carried out since there were no similar validated instruments that measure the perception of university students towards learning; we only knew the dimensions that make up the construct according to the literature consulted.

To do so, firstly the data were assessed obtaining significant values both in the Kaiser-Meyer-Olkin test, (KMO= 0.941) and the Bartlett’s sphericity test (*c* ^2^= 2446.206; df= 300; *p* < 0.001). These values indicate that a factorial analysis was an adequate technique for interpreting the information contained in this matrix. This analysis yielded three clearly defined factors that explain 61.61% of the total variance. The results of the CFA confirm the three-factor model. The indexes of goodness used were χ^2^/df =1.944 (which is within the established standards), the RMR is 0.077 and the RMSEA is 0.080, which are considered acceptable as they are between 0.5 and 0.08^(^ 49 ^)^. In addition, the GFI representing the joint adjustment is 0.78 and CFI is 0.97, so both values are within the tolerance limits. These results confirm that the data fit to the 3-factor model, supporting the proposed dimensional structure.

For all these reasons, these results allow us to be confident in the reliability and validity of this instrument. Therefore, the construction and validation of this questionnaire make possible the application of this instrument to measure the student’s perception in the use of IRS in the learning process.

Regarding the factor “Learning environment”, reliability was measured using the Cronbach’s alpha coefficient, which scores 0.926 and suggests a high internal consistency. The EFA explains 51.7% of the total variance, being the factor with the highest punctuation in this questionnaire. Among the items comprising this dimension are: item 15: the use of IRS makes the classes enjoyable and dynamic, item 48: the use of *IRS improves my participation in the classes behind anonymity, item 47: the use of IRS makes the classes more enjoyable and interactive compared to traditional classes*, among others. This factor is decisive since university students with an adequate class environment increase class attendance^(^ 22 ^)^ and improves their participation^(^ 13 ^,^ 17 ^,^ 44 ^)^. It also comes on interaction between professors and students^(^ 13 ^)^, and has a positive influence on attention^(^ 33 ^-^ 34 ^)^ and concentration^(^ 35 ^)^, as the studies analyzed show.

The second dimension, which corresponds to the “teaching-learning process” factor, shows an internal consistency index of 0.869 and explains 5.4% of the total variance, significantly lowering the weight of the factor. This dimension refers to those elements that are basic to acquire knowledge, such as debates and interaction between professors and students, which positively affects their learning process since it helps students to review and understand the contents. Among the items that make up this factor are: item 47 *the use of IRS makes the classes more pleasant and interactive in comparison with traditional classes, item 59 IRS provides valuable information to improve my learning process, item 46 the use of IRS is done at the end of the classes to review the contents explained during the session*.

These elements coincide with studies supporting that this working methodology improves performance^(^ 31 ^-^ 32 ^,^ 38 ^-^ 41 ^)^ thanks to the paradigm shift that implies active learning through the connection between knowledge and know-how^(^ 45 ^)^ and the frequent and positive interaction, which resulted into more dynamic classes when IRS is used^(^ 13 ^,^ 28 ^)^. This method promotes material comprehension by acquiring a deeper knowledge, helping to review and understand the contents and improving long-term retention^(^ 36 ^-^ 37 ^)^; the final result is an improvement in the learning process.

In relation to the third dimension, “Learning Assessment” presents a Cronbach’s alpha of 0.871, and the EFA explains 5.07% of the total variance, showing a similar percentage to the previous factor. This dimension is related to feedback and formative evaluation, which help to correct errors or misunderstandings about the contents of the subject worked on^(^ 4 ^,^ 20 ^,^ 40 ^)^. The items included in this factor are: item 20 the use of *IRS allows you to know and compare your colleagues’ answers with your own answers; item 36 the use of IRS promotes regular study of the subject to be better prepared for classes; item 35, The use of IRS evaluates my comprehensive knowledge of the contents in each of the topics covered during the classes*. The immediate feedback has a positive impact on both the students in their learning process and the professor in their teaching process, driving the students’ formative evaluation.

In this way, in our global context with no academic frontiers, the European and Latin American Higher Education areas meet the premise that university system must be in a continuous transformation towards active student learning and lifelong learning. In addition, it is necessary to establish evaluation and accreditation of the work carried out in universities, promoting the transference of knowledge between successive researches among different fields of knowledge. This is the way an interdisciplinary understanding can be developed to assess the reality and to question the traditional consideration of the fields of knowledge as isolated compartments separated by disciplinary boundaries.

Among the limitations of this research, we list the following: firstly, it is necessary to continue increasing the number of participants, since the sample is not excessively large, but it does provide a starting point for transferring it to other fields of knowledge. The reason for this is the limited number of professors at the Melilla Campus of the University of Granada who use technopedagogical resources such as IRS in their classrooms and integrate them to processes of active gamification methodology, which is why it is necessary to promote and explore these resources. It is therefore essential to raise awareness and train university professors in the TPACK (Technological Pedagogical and Content Knowledge) model as a conceptual framework that can guide professors when integrating technology into students’ learning processes, which will promote emerging methodologies such as gamification.

Secondly, improving the instrument is crucial so that it can be used by professors in any field of knowledge and in any situation (as has been done during the COVID-19 confinement). The objective is launching an instrument for future periods, according to the Government and university approaches, that can be used in face-to-face training, B-Learning or E-Learning. Therefore, the instrument needs to be developed by including new items that assess this new virtual education models. In addition, we must deepen the influence of this instrument depending on the different disciplines of the Health Sciences, a research that we intend to carry out in the future. The application of technological advances in the university field comes on the active learning process of students, and it is necessary to know their opinion to achieve progress and improvement in teaching. Thirdly, it is necessary further quasi-experimental research in the several fields of university teaching to analyze the influence of gamified active methodology through these technopedagogical resources and traditional methodology, bearing in mind its scope or relationship with academic performance.

## Conclusion

The contribution of this study to knowledge is the design, development and dissemination of a new instrument that allows the measurement and assessment of students’ perception on the use of IRS during university training by using a technopedagogical resource in any field of knowledge (whether Health Sciences, Social and Legal Sciences, Arts and Humanities, or Engineering and Architecture). The instrument considers three fundamental factors: learning environment, teaching-learning process and assessment. This questionnaire can be applied in the classroom during the training, helping to improve the teaching-learning processes. In this sense, Health Sciences professors must establish and explore innovative ways to involve students and stimulate active learning. It is important to incorporate active methods by using interactive response commands in disciplines such as nursing, medicine, pharmacy, paramedical education, psychology, dentistry, physiotherapy, speech therapy, biotechnology, epidemiology, genetics, biochemistry, occupational therapy, human nutrition and dietetics, among others. This instrument provides a positive pedagogical approach in the teaching-learning process for Health Sciences professors and students, improving the academic performance with the purpose of acquiring a deep knowledge of the subjects.
